# Berberine enhances the function of db/db mice islet β cell through GLP-1/GLP-1R/PKA signaling pathway in intestinal L cell and islet α cell

**DOI:** 10.3389/fphar.2023.1228722

**Published:** 2023-07-04

**Authors:** Wenbin Wu, Qingsong Xia, Yujin Guo, Hongzhan Wang, Hui Dong, Fuer Lu, Fen Yuan

**Affiliations:** ^1^ Institution of Integrated Traditional Chinese and Western Medicine, Tongji Hospital, Tongji Medical College, Huazhong University of Science and Technology, Wuhan, Hubei, China; ^2^ Department of Integrated Traditional Chinese and Western Medicine, Tongji Hospital, Tongji Medical College, Huazhong University of Science and Technology, Wuhan, Hubei, China

**Keywords:** berberine, GLP-1, GLP-1r, islet β cell, T2DM

## Abstract

**Background:** The evidence on berberine stimulating the secretion of GLP-1 in intestinal L cell has been studied. However, few research has explored its role on generating GLP-1 of islet α cell. Our experiment aims to clarify the mechanism of berberine promoting the secretion of GLP-1 in intestinal L cell and islet α cell, activating GLP-1R and its downstream molecules through endocrine and paracrine ways, thus improving the function of islet β cell and treating T2DM.

**Methods:** After confirming that berberine can lower blood glucose and improve insulin resistance in db/db mice, the identity maintenance, proliferation and apoptosis of islet cells were detected by immunohistochemistry and immunofluorescence. Then, the activation of berberine on GLP-1/GLP-1R/PKA signaling pathway was evaluated by Elisa, Western blot and PCR. Finally, this mechanism was verified by *in vitro* experiments on Min6 cells, STC-1 cells and aTC1/6 cells.

**Results:** Berberine ameliorates glucose metabolism in db/db mice. Additionally, it also increases the number and enhances the function of islet β cell. This process is closely related to improve the secretion of intestinal L cell and islet α cell, activate GLP-1R/PKA signaling pathway through autocrine and paracrine, and increase the expression of its related molecule such as GLP-1, GLP-1R, PC1/3, PC2, PKA, Pdx1. *In vitro*, the phenomenon that berberine enhanced the GLP-1/GLP-1R/PKA signal pathway had also been observed, which confirmed the results of animal experiments.

**Conclusion:** Berberine can maintain the identity and normal function of islet β cell, and its mechanism is related to the activation of GLP-1/GLP-1R/PKA signal pathway in intestinal L cell and islet α cell.

## Introduction

Type 2 diabetes (T2DM) has become one of the biggest health crises. Recent data reported by the International Diabetes Federation (IDF) in 2021 have demonstrated that the global prevalence of diabetes was estimated at 10.5% (approximately 537 million people) (Sun H. et al., 2022). Patients with T2DM are usually older than 65 years old, with varying degrees of potential insulin resistance and uncontrolled hyperglycemia (Perreault et al., 2021). T2DM is due to insulin resistance and β cell dysfunction, gradually leading to uncontrollable hyperglycemia (Rizza, 2010). In the T2DM process, the compensatory high insulin secretion state of β cell will lead to the dedifferentiation and transdifferentiation of β cell (Hudish et al., 2019). As the duration of T2DM prolongs, the dysfunction of β cell will also deepen (Sun et al., 2019).

Glucagon-like peptide-1 (GLP-1) is an insulin-promoting hormone and can enhance glucose-dependent insulin secretion, inhibit glucagon secretion and delay gastric emptying. In addition, GLP-1 can also inhibit the identity loss and dysfunction of pancreatic islets β cell (Muller et al., 2019). GLP-1 agonists are widely used in the treatment of T2DM and have significant therapeutic effects on patients with hyperglycemia, insulin resistance, and β cell dysfunction (Lee et al., 2018). In the past, it was recognized that it was produced by intestinal L cell, but now it is found that pancreatic α cell can also secrete GLP-1. Functional supplementation by islet derived GLP-1 when intestinal derived GLP-1 secretion decreases (Marchetti et al., 2012). GLP-1 acts on GLP-1R of pancreatic β cell through endocrine and paracrine ways respectively (Huising, 2020). GLP-1R activates adenylate cyclase (AC) to produce cAMP, which in turn activates protein kinase A (PKA) (Que et al., 2019).

PC1/3 and PC2 are neuroendocrine specific endoproteases belonging to the subtilisin-like serine protease family. They can process peptide hormones into bioactive products (Smeekens et al., 1992). Under their shearing action, proinsulin is converted into insulin and glucagon and secreted into blood. At the same time, PC1/3 has the effect of cutting glucagon to form GLP-1 (Muller et al., 2019). Pdx1 is a major regulator of pancreatic organogenesis, β cell maturation and identity preservation, and also has the function of promoting normal insulin secretion (Ebrahim et al., 2022). Research has found that after high glucose and high fat culture *in vitro*, the expression of Pdx1 in primary pancreatic islets decreases, intervention with liraglutide can significantly increase the secretion of Pdx1, while GLP-1R inhibitors can counteract this effect (Cataldo et al., 2021). The GLP-1/GLP-1R/PKA signaling pathway can maintain the identity of islet β cell and promote insulin secretion by promoting the expression of these molecules.

Berberine is a bioactive component of huanglian (Coptis chinensis Franch.), which has extensive pharmacological effects and is widely used in the treatment of digestive and endocrine system diseases in clinical practice (Song et al., 2020). When studying the mechanism of berberine in the treatment of diabetes, a study has already pointed out that berberine can promote insulin secretion, improve insulin resistance and inhibit pancreatic islet β cell dysfunction (He Q. et al., 2022), Meanwhile, a study has also confirmed that berberine promotes the secretion of GLP-1 by L cell in intestinal tissue, while activating GLP-1R and the phosphorylation of PKA through endocrine pathways (Sun S. et al., 2022). However, there is relatively little or no research on the protection of pancreatic islet cell identity and promotion of GLP-1 parasecretion by berberine. Our study supplements the mechanism by which berberine protects the identity and function of pancreatic islet cells from the perspective of promoting GLP-1 paracrine secretion by islet α cell.

In this experiment, we used *in vivo* and *in vitro* experiments and explored the process of berberine improving the function of islet β cell by stimulating the endocrine and paracrine GLP-1 in intestinal L cell and islet α cell and activating the GLP-1R/PKA signaling pathway.

## Materials and methods

### Antibodies and reagents

PC2 antibody (D1E1S), t-PKA antibody (D38C6), p-PKA antibody (D45D3), Pdx1 antibody (D59H3), GLP-1R antibody (HA500204) and all secondary antibodies used in Western blot were purchased from Cell Signaling Technology (Beverly, MA, United States); PC1/3 antibody (ab220363) was obtained from Abcam (Cambridge, United Kingdom); Ki67 antibody (A16919) was obtained from Abclonal (Wuhan, China); GLP-1 antibody (55292-1-AP), insulin antibody (66198-1-Ig) and glucagon antibody (15954-1-AP) were bought from Proteintech (Wuhan, China); β-actin antibody (20536-1-AP) was obtained from Santa CruzBiotechnology (Santa Cruz, CA). Berberine, Palmitic acid (PA), GLP-1 (9-36) amide were purchased from Sigma-Aldrich (MO, United States). Other regular reagents were obtained from Wuhan Gugeshengwu Technology Co., Ltd. unless otherwise specified.

### Animal experiment

**FIGURE 1:**
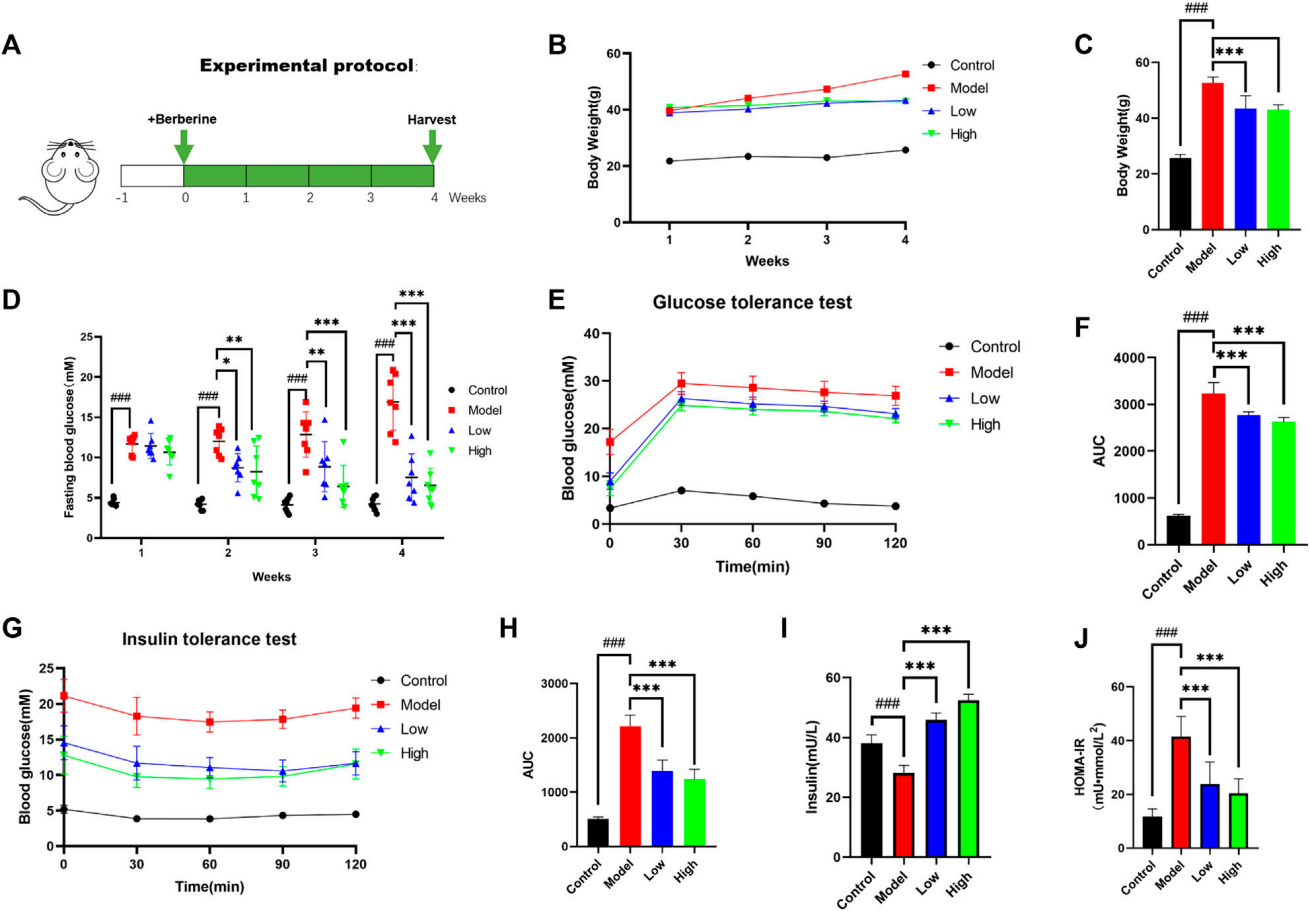
Berberine improved T2DM symptoms in db/db mice. **(A)** Animal experimental protocol. **(B)** Body weight of mice was recorded weekly (*n* = 8). **(C)** The bar graph represents the final body weight at the ending of experiment (*n* = 8). **(D)** Fasting blood glucose of mice was recorded weekly (*n* = 7). **(E)** For GTT, mice were fasted overnight and intraperitoneally injected with glucose (i.p., 0.75 g/kg), blood glucose was measured at 0, 30, 60, and 120 min after glucose adminis-tration (*n* = 7). **(F)** The bar graph represents average area under the GTT curve (*n* = 7). **(G)** For ITT, mice were fasted overnight and intraperitoneally injected with insulin (i.p., 1.0 U/kg), blood glucose was measured at 0, 30, 60, and 120 min after insulin administration; the bar graph represents average area under the curve (*n* = 7). **(H)** The bar graph represents average area under the ITT curve (*n* = 7). **(I)** Fasting insulin was determined at the ending of experiment (*n* = 5). **(J)** HOMA-IR index was calculated according to standard formula: HOMA-IR = FBG (mM) × fasting insulin (mU/L)/22.5 (*n* = 8). All data are presented as means ± SEM. Compared to control group, #*p* < 0.05, ##*p* < 0.01, ###*p* < 0.001; Compared to model group, *p < 0.05, **p < 0.01, ***p < 0.001.

Male seven-week-old db/db and db/m mice were obtained from Nanjing Biomedical Research Institute of Nanjing University. We kept the animals in the animal experiment center (SPF-grade) of Huazhong University of science and technology (22°C±2°C, 12 h light/dark cycles, 40%–60% humidity). Db/m mice were set as the Control group, and db/db mice were randomly divided into Model group, Low group and High group. The Control group and Model group were given distilled water by gavage, while the Low group and High group were given 150 mg/kg and 300 mg/kg berberine by gavage respectively. The experimental protocol is illustrated in Figure 1A. After the experiment, 3% pentobarbital sodium (45 mg/kg, intraperitoneal injection) was used for anesthesia. The separated pancreas and ileum were partially fixed in polyformaldehyde and partially placed in a refrigerator at −80°C. The animal experiment was approved by the Animal Ethics Committee of Tongji Medical College, Huazhong University of Science and Technology (HUST) and its IACUC number is S2095.

### Measurement of insulin and GLP-1

Insulin and GLP-1 was quantified by ELISA kits. We drew a standard curve using the gradient dilution of the standard product as a template, added the sample, antibody and HRP aggregation reagent, and put it in a 37°C water bath, then washed it five times with a washing solution, we added the chromogenic solution and reacted for 10 min, then detected the absorbance of each hole at a wavelength of 450 nm.

### Immunohistochemistry and immunofluorescence staining

For immunohistochemistry, paraffin sections were dewaxed and washed. Then, the slices were placed in the microwave to repair the antigen. After cooling to room temperature, the chips were put into 3% H_2_O_2_ for 30 min. After washing with PBS, chips were sealed with 10% goat serum for 1 h. The first antibody was added and incubated at 4°C for 14 h and the second antibody was added and incubated for 1 h. DAB is used for color development, and then hematoxylin is used for nuclear staining.

For immunofluorescence, except for the need to block endogenous peroxidase with H_2_O_2_, its process did not differ from immunohistochemistry until the primary antibody was incubated. After the primary antibody was incubated for 14 h, the secondary antibody was added and incubated for 1 h. We used DAPI to stain the nucleus.

### Cell culture

The mouse pancreatic β-cell line, Min6, was cultured in DMEM high glucose medium containing 10% fetal bovine serum. The mouse intestinal L-cell line, STC-1, was cultured in 1648 medium containing 10% fetal bovine serum. The mouse pancreatic α-cell line, αTC1/6, was cultured in DMEM high glucose medium containing 15% fetal bovine serum.

PA was used to construct T2DM model in cells, and berberine was used to observe its therapeutic effect. GLP-1 (9-36) amide was a competitive inhibitor of GLP-1R, and was used to observe the effect of berberine on GLP-1/GLP-1R/PKA signal pathway.

After determining the appropriate drug concentration in each cell with cck8, the three drugs were applied to the cell for 24 h. The culture supernatants were collected for GLP-1 assays, and the cells were lysed for protein or mRNA analysis.

### Western blot analysis

Proteins were extracted from tissues and cells, and then protein concentrations were quantified using a Bis-creatine (BCA) kit. Samples were electrophoresed (80 V, 0.5 h, then 120 V, 1H) and transferred to polyvinylidene fluoride membranes (280 mA, 1 kda/min). Bands were put into blocking buffer for 1 h and incubated in primary antibody for 14 h. After cleaning, the secondary antibody was applied for 1 h. After cleaning, the strips were sent for exposure.

### Real-time quantitative polymerase chain reaction (RT-qPCR)

The samples were added into Trizol for cracking, then chloroform was added for centrifugation to obtain the upper liquid phase, and isopropyl alcohol was added for centrifugation to obtain the precipitate. The precipitate was washed with ethanol and then dissolved with DEPC water. After reverse transcription, reagents and sample cDNA were added according to PCR reaction system and performed on LightCycler^®^96 system (Roche Diagnostics, Mannheim, Germany). Sequences of the primers are listed in Table 1.

**TABLE 1:** Primers used for RT-qPCR.

Gene	Forward (5′–3′)	Reverse (5′–3′)
GLP-1R	CTC​CGA​GCA​CTG​TCC​GTC​TT	GAT​AAC​GAA​CAG​CAG​CGG​AAC
PC1/3	GTA​CTG​TTG​GCT​GAA​AGG​GAA​AG	CGC​TTC​TCC​ACA​ACA​TTC​ACC
PC2	TCT​TGA​CCT​ACG​GCA​TGA​TGA​G	CAC​TCC​TAG​CAG​CAG​GTT​CTC​AT
Pdx1	AGC​TCG​CTG​GGA​TCA​CTG​GA	TGT​AAG​CAC​CTC​CTG​CCC​ACT
GAPDH	CCT​CGT​CCC​GTA​GAC​AAA​ATG	TGA​GGT​CAA​TGA​AGG​GGT​CGT

### Statistical analysis

Data are presented as mean ± SD. All data were tested for normality using Shapiro wilk. When *p* < 0.05, we performed Kruskal–Wallis analysis. When *p* > 0.05, we used one-way ANOVA to compare differences between groups. Statistical analysis was performed using GraphPad Prism software, and *p* < 0.05 was considered statistically significant.

## Results

### Berberine ameliorates diabetic conditions in db/db mice

As a mature T2DM animal model, db/db mice were used to explore the effect of berberine on their T2DM symptoms. We recorded the weight of mice since the beginning of the experiment and the differences in the last weight of each group. Compared with the Model group, the Low group and the High group had a clear weight loss effect (Figure 1B). We recorded the changes of the fasting blood glucose level of mice every week. We can see that the rate of fasting blood glucose decline in the Low group is slightly slower than that in the High group, but it also maintained at a lower level with the High group at the end of the experiment (Figures 1C, D). After the administration of berberine, we conducted glucose tolerance test (GTT) and insulin tolerance test (ITT). Compared with db/db mice, the Low and High groups treated with berberine had lower blood glucose levels after intraperitoneal injection of glucose and insulin (Figures 1E, G). At the same time, the area under the curve (AUC) of GTT and ITT in these two groups was also lower (Figures 1F, H). In order to evaluate the effect of berberine on insulin secretion, we measured the serum insulin level at fasting. It can be found that compared with the Model group, the Low group and High group secreted more insulin after berberine treatment (Figure 1I), and calculated homeostatic model assessment of insulin resistance (HOMA-IR) index according to the following formula: HOMA-IR = FBG (mM) × Fasting insulin (mU/L)/22.5 (Figure 1J). These data suggested that berberine ameliorates diabetic conditions in db/db mice.

### Effect of berberine on islet cells of db/db mice

**FIGURE 2:**
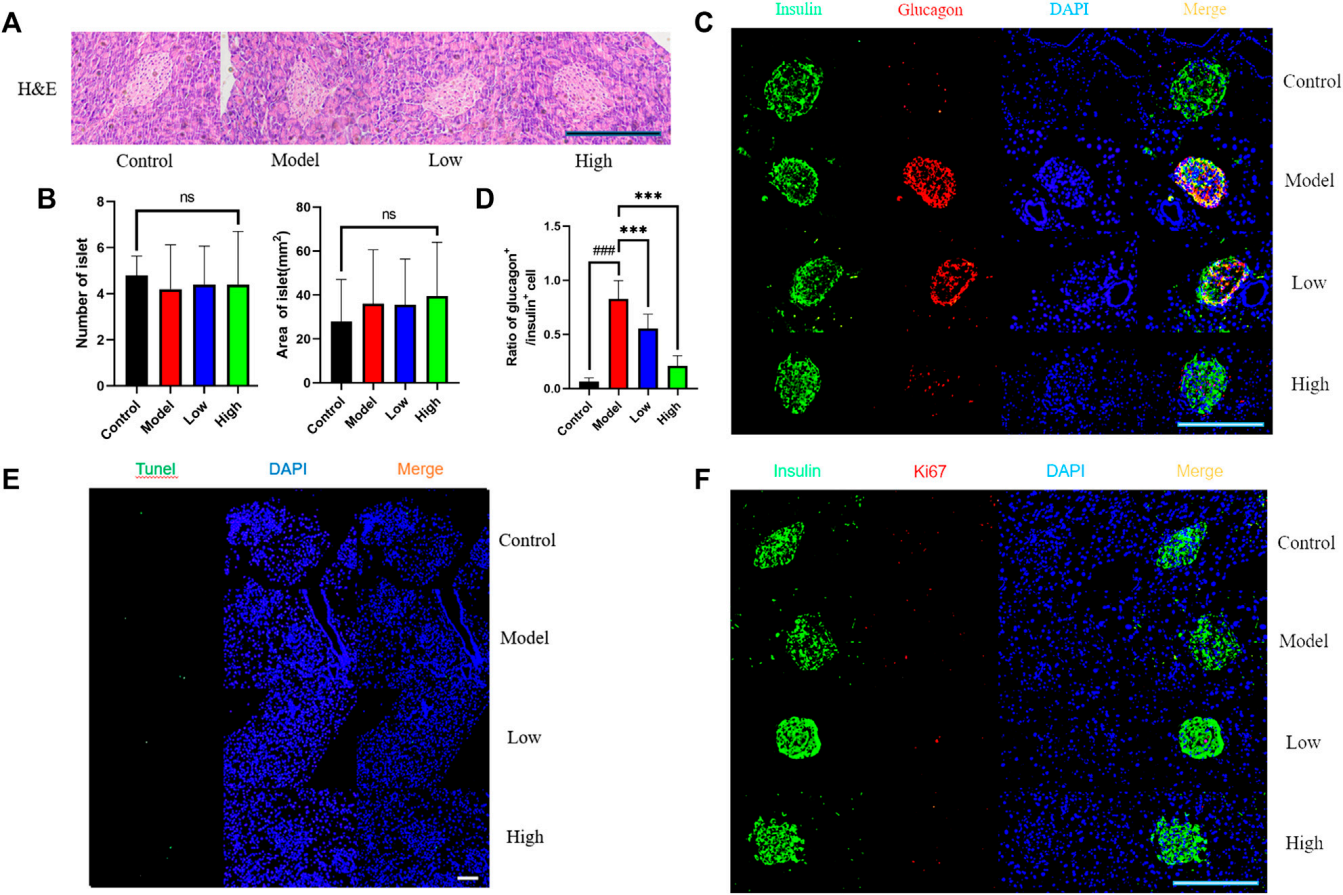
Effect of berberine on islet cells of db/db rats. **(A)** Representative H&E staining in different groups of pancreas. Scale bar, 400 μm. **(B)** Representative figure of islet number and islet area in different groups (*n* = 5). **(C)** Immunofluorescence images showing the glucagon (red) and insulin (green) expression in different groups of pancreas. DAPI staining indicates the nuclei (blue). Scale bar, 400 μm. **(D)** Representative figure of the ratio of glucagon^+^ cells to insulin^+^ cells in (*n* = 3). **(E)** Representative figures of TUNEL (green) immunofluorescence staining in different groups of pancreas. DAPI staining indicates the nuclei (blue). Scale bar, 400 μm. **(F)** Immunofluorescence images showing the Ki67 (red) and insulin (green) expression in different groups of pancreas. DAPI staining indicates the nuclei (blue). Scale bar, 400 μm. All data are presented as means ± SEM. Compared to control group, #*p* < 0.05, ##*p* < 0.01, ###*p* < 0.001; Compared to model group, *p < 0.05, **p < 0.01, ***p < 0.001.

We used H&E staining method to observe the shape of mouse islets (Figure 2A). After comparing the shape, quantity and area of islets, that there was no significant difference in these indicators between groups (Figure 2B). We used glucagon and insulin to label α cell and β cell for co-staining, and found that the ratio of α cell to β cell in Model group increased observably, while the two groups treated with berberine improved this trend (Figure 2C, D). Then we used Tunel fluorescence staining to detect the apoptosis signal in islet cells and immunofluorescence staining for the value-added related antigen Ki67 (Figures 2E, F). However, the expression of value-added and apoptosis-related signals between the groups was very little, and there was no significant difference. These data indicate that berberine had no significant effect on the morphology, quantity and proliferation apoptosis of islet cells, but it inhibited db/db mice β cell transformation α cell process. Therefore, we had further studied the signal pathway that maintains the identity of β cell and inhibits β cell dysfunction.

### Berberine increased the expression of GLP-1 and GLP-1R

**FIGURE 3:**
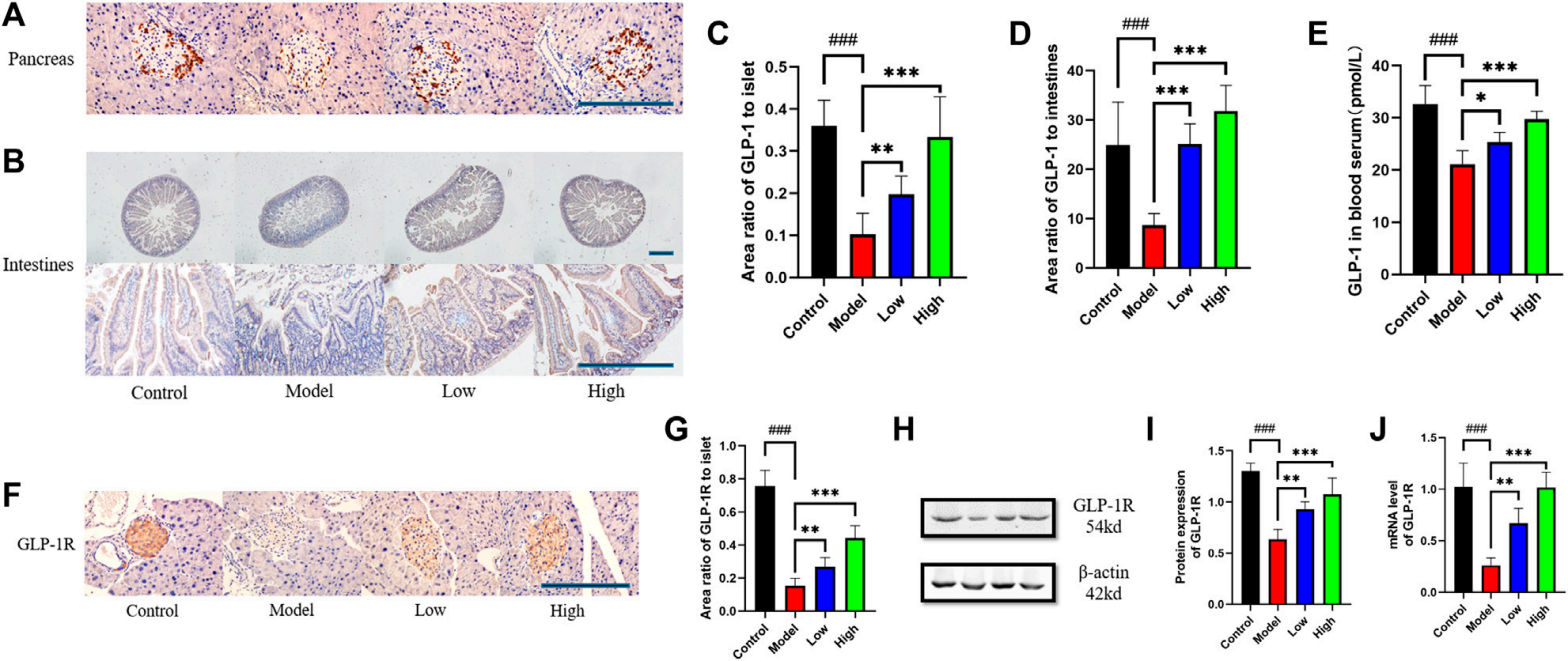
Berberine increased the expression of GLP-1 and GLP-1R. **(A)** Representative immunohistochemical figures of GLP-1 in pancreas. Scale bar, 400 μm. **(B)** Representative immunohistochemical figures of GLP-1 in intestine. Scale bar, 400 μm. **(C)** Representative figure of GLP-1 area in pancreas (*n* = 3). **(D)** Representative figure of GLP-1 area in intestine (*n* = 5). **(E)** Representative figure of GLP-1 in blood serum (*n* = 5). **(F)** Representative immunohistochemical figures of GLP-1R in intestine. Scale bar, 400 μm. **(G)** Representative figure of GLP-1 area in pancreas (*n* = 3). **(H)** Representative western blots for immunoprecipitation of GLP-1R in intestine. **(I)** The quantification of GLP-1R immunoprecipitation in intestine (*n* = 4). **(J)** The mRNA levels of GLP-1R in intestine of different groups (*n* = 4). All data are presented as means ± SEM. Compared to control group, #*p* < 0.05, ##*p* < 0.01, ###*p* < 0.001; Compared to model group, *p < 0.05, **p < 0.01, ***p < 0.001.

The expression level of GLP-1 in pancreas and intestine was measured by immunohistochemistry. The pancreas was photographed under ×400 microscope and the intestine was photographed under ×100 and ×400 microscope respectively (Figures 3A, B). Through statistical analysis of GLP-1 positive area, it can be seen that the expression of GLP-1 in the pancreas and intestine of the Model group decreased compared with that of the Control group, while the Low and High groups given berberine reversed this trend (Figures 3C, D). In addition to GLP-1 in tissues, we used ELISA to detect the level of GLP-1 in serum, and its trend was also consistent with that of GLP-1 in pancreas and intestine (Figure 3E). We observed GLP-1R in the pancreas under ×400 microscope by immunohistochemistry, and found that berberine reversed the downward trend of GLP-1R expression in the Model group (Figures 3F, G). For the expression of GLP-1R in the intestine, we used Western blot to detect its protein content and PCR to detect its transcription level (Figure 3H). Statistics showed that berberine increased the expression of GLP-1R in db/db mice at the protein content and mRNA level (Figures 3I, J). These data indicated that berberine can activate the intestinal—islet GLP-1/GLP-1R/PKA signaling pathway.

### Berberine increased the expression of PKA, PC1/3, PC2, and Pdx1

**FIGURE 4:**
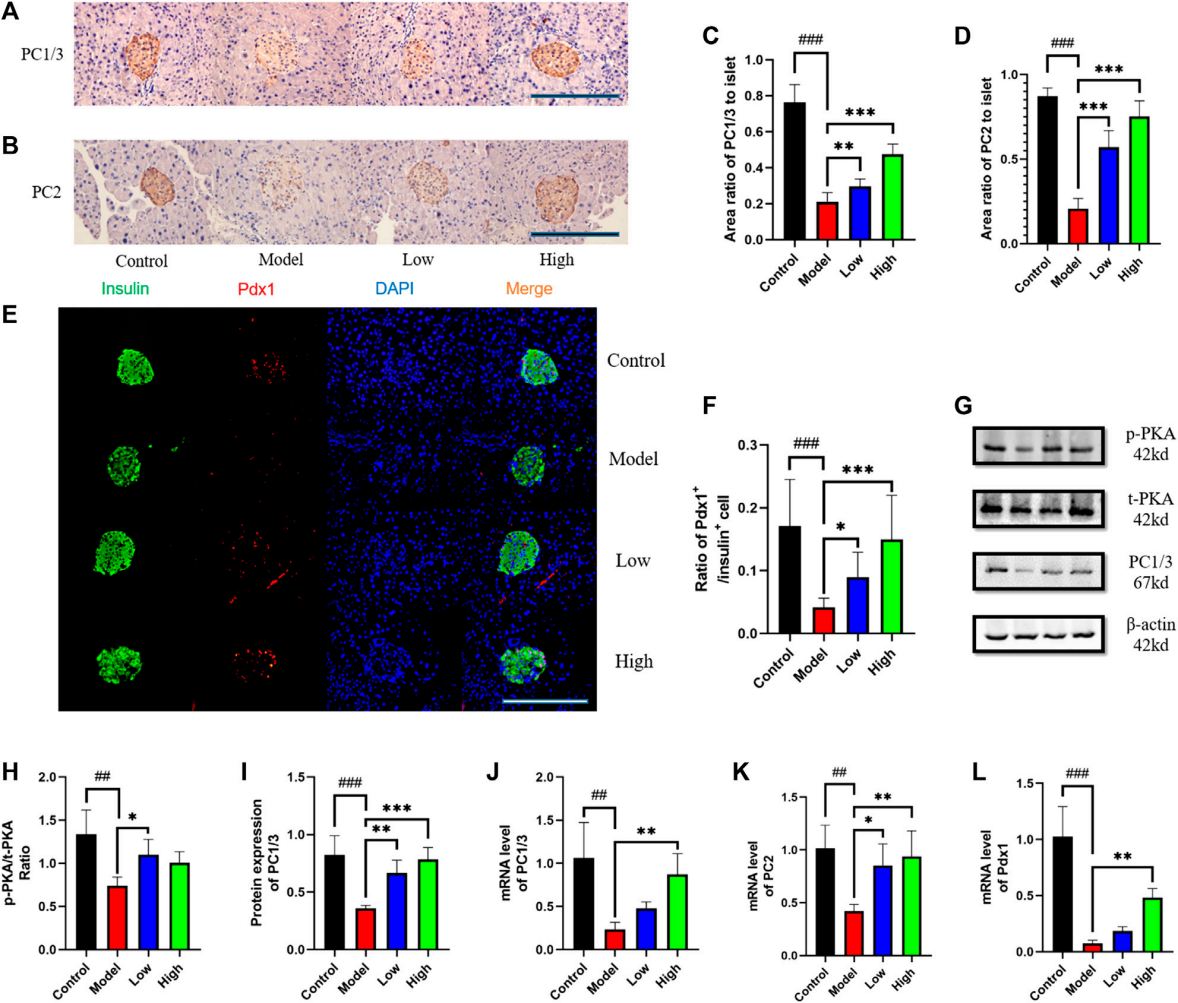
Berberine increased the expression of PKA, PC1/3, PC2, and Pdx1. **(A)** Representative immunohistochemical figures of PC1/3 in pancreas. Scale bar, 400 μm. **(B)** Representative immunohistochemical figures of PC1/3 in pancreas. Scale bar, 400 μm. **(C)** Representative figure of PC1/3 area in pancreas (*n* = 3). **(D)** Representative figure of PC2 area in pancreas (*n* = 3). **(E)** Immunofluorescence images showing the Pdx1 (red) and insulin (green) expression in different groups of pancreas. DAPI staining indicates the nuclei (blue). Scale bar, 400 μm. **(F)** Representative figure of the ratio of Pdx1^+^ cells to insulin^+^ cells in different groups of pancreas (*n* = 3). **(G)** Representative western blots for immunoprecipitation of PKA and PC1/3 in intestine. **(H)** The quantification of PKA immunoprecipitation in intestine (*n* = 4). **(I)** The quantification of PC1/3 immunoprecipitation in intestine (*n* = 4). **(J)** The mRNA levels of PC1/3 in intestine of different groups (*n* = 4). **(K)** The mRNA levels of PC2 in intestine of different groups (*n* = 4). **(L)** The mRNA levels of Pdx1 in intestine of different groups (*n* = 4). All data are presented as means ± SEM. Compared to control group, #*p* < 0.05, ##*p* < 0.01, ###*p* < 0.001; Compared to model group, *p < 0.05, **p < 0.01, ***p < 0.001.

In the pancreas, we observed PC1/3 and PC2 under X400 microscope by immunohistochemical method (Figures 4A, B). Statistics showed that PC1/3 and PC2 in Model group was significantly lower than that in Control group, and then increased after berberine treatment (Figures 4C, D). We observed the expression level of Pdx1 by immunofluorescence method and found that it increased after berberine treatment (Figures 4E, F). In the intestine, we detected the protein levels of PC1/3 and PKA by Western blot (Figure 4G), and found that the expression of PC1/3 and the phosphorylation level of PKA increased after the treatment of berberine (Figures 4H, I). At the same time, we detected the mRNA levels of PC1/3, PC2 and Pdx1. The treatment of berberine promoted the transcription of these molecules in the intestine (Figures 4J–L). These data indicated that the status of β cell in pancreas and intestine is maintained and the molecular expression level of β cell dysfunction is increased after treatment.

### Berberine activated GLP-1/GLP-1R/PKA signaling pathway in Min6 cell

**FIGURE 5:**
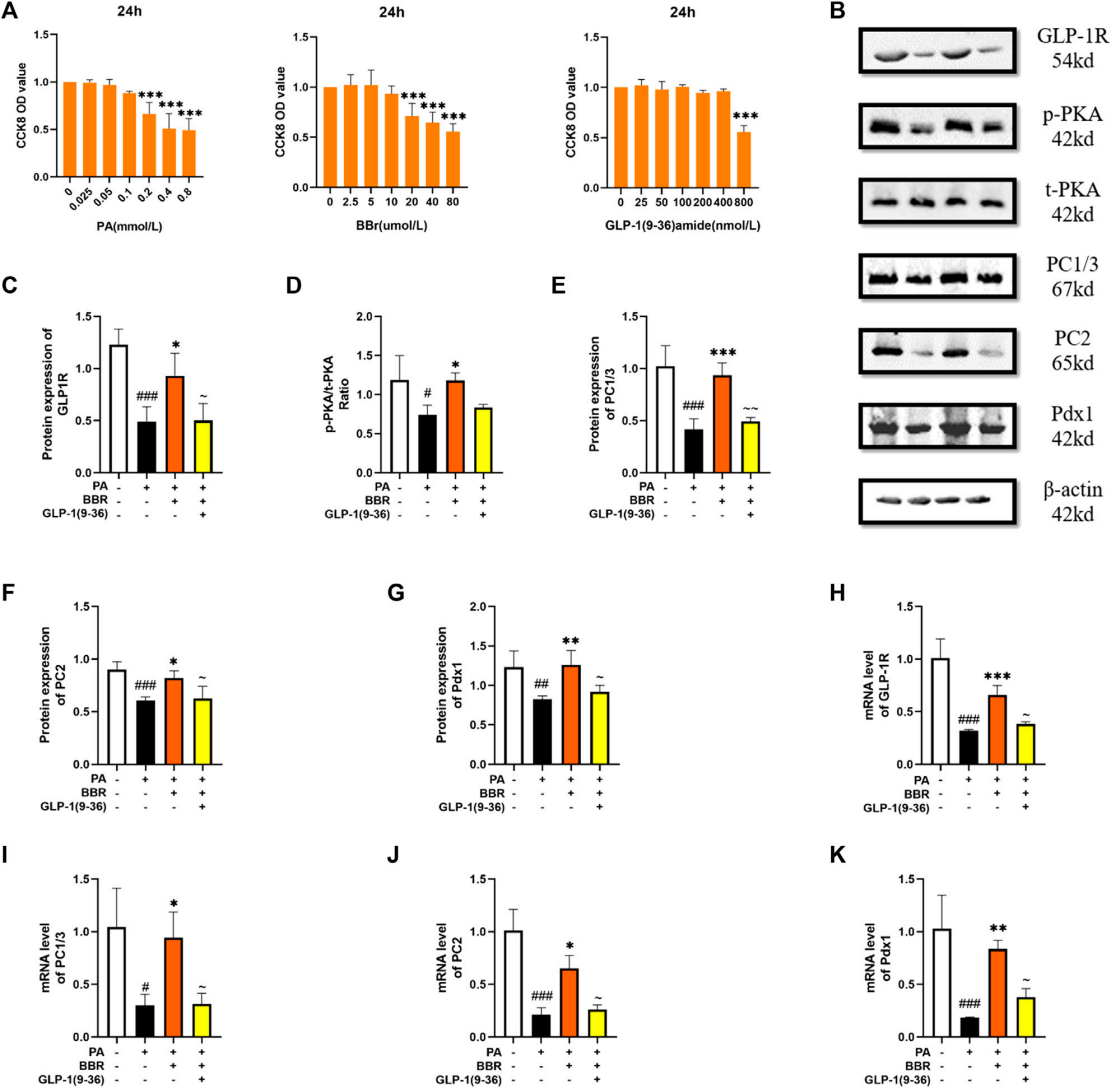
Berberine activated GLP-1/GLP-1R/PKA signaling pathway in Min6 cells. **(A)** The cck8 representative figures of PA, BBR and GLP-1 (9-36) amide (*n* = 3). **(B)** Representative western blots for immunoprecipitation of GLP-1R, PKA, PC1/3, PC2 and Pdx1 in Min6. **(C)** The quantification of GLP-1R immunoprecipitation in Min6 (*n* = 4). **(D)** The quantification of PKA immunoprecipitation in Min6 (*n* = 4). **(E)** The quantification of PC1/3 immunoprecipitation in Min6 (*n* = 4). **(F)** The quantification of PC2 immunoprecipitation in Min6 (*n* = 4). **(G)** The quantification of Pdx1 immunoprecipitation in Min6 (*n* = 4). **(H)** The mRNA levels of GLP-1R in Min6 of different groups (*n* = 3). **(I)** The mRNA levels of PC1/3 in Min6 of different groups (*n* = 3). **(J)** The mRNA levels of PC2 in Min6 of different groups (*n* = 3). **(K)** The mRNA levels of Pdx1 in Min6 of different groups (*n* = 3). All data are presented as means ± SEM. Compared to control group, #*p* < 0.05, ##*p* < 0.01, ###*p* < 0.001; Compared to model group, *p < 0.05, **p < 0.01, ***p < 0.001; Compared to BBR group, ∼*p* < 0.05, ∼∼*p* < 0.01, ∼∼∼*p* < 0.001.

Min6 cell is a cell line established in insulinoma obtained by targeted expression in transgenic mice, and retains the physiological characteristics of normal β cell. We used the method of CCK8 to determine the concentration of PA, berberine and GLP-1 (9-36) amide in Min6 cell (Figure 5A). Then, protein content of GLP-1R, PKA, PC1/3, PC2 and Pdx1 in four groups of cells was detected using Western blot (Figure 5B). After statistics, we found that the expression of these proteins decreased after PA modeling, and increased after adding berberine, while after giving GLP-1R inhibitor, this trend of berberine was inhibited (Figures 5C–G). We measured mRNA levels of GLP-1R, PC1/3, PC2 and Pdx1 in cells by PCR, and the trend was also consistent with the trend of their protein expression (Figures 5H–K). These data indicated that berberine activated the GLP-1/GLP-1R/PKA signaling pathway in Min6 cell.

### Berberine activated GLP-1/GLP-1R/PKA signaling pathway in STC-1 cell

**FIGURE 6:**
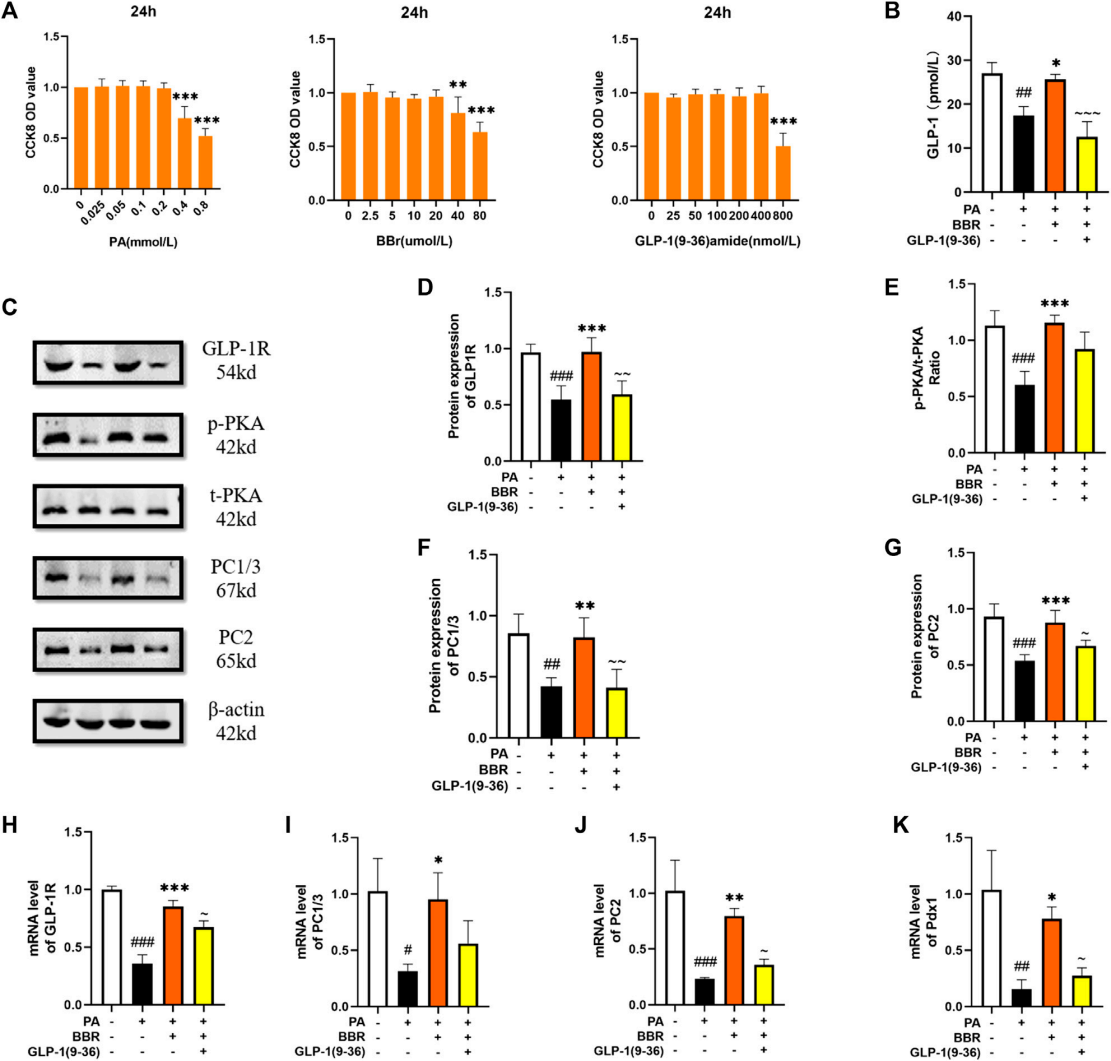
Berberine activated GLP-1/GLP-1R/PKA signaling pathway in STC-1 cells. **(A)** The cck8 representative figures of PA, BBR and GLP-1 (9-36) amide (*n* = 3). **(B)** Representative figure of GLP-1 in cell (*n* = 3). **(C)** Representative western blots for immunoprecipitation of GLP-1R, PKA, PC1/3 and PC2 in STC-1. **(D)** The quantification of GLP-1R immunoprecipitation in STC-1 (*n* = 4). **(E)** The quantification of PKA immunoprecipitation in STC-1 (*n* = 4). **(F)** The quantification of PC1/3 immunoprecipitation in STC-1 (*n* = 4). **(G)** The quantification of PC2 immunoprecipitation in STC-1 (*n* = 4). **(H)** The mRNA levels of GLP-1R in STC-1 of different groups (*n* = 3). **(I)** The mRNA levels of PC1/3 in STC-1 of different groups (*n* = 3). **(J)** The mRNA levels of PC2 in STC-1 of different groups (*n* = 3). **(K)** The mRNA levels of Pdx1 in STC-1 of different groups (*n* = 3). All data are presented as means ± SEM. Compared to control group, #*p* < 0.05, ##*p* < 0.01, ###*p* < 0.001; Compared to model group, *p < 0.05, **p < 0.01, ***p < 0.001; Compared to BBR group, ∼*p* < 0.05, ∼∼*p* < 0.01, ∼∼∼*p* < 0.001.

STC-1 cell has many characteristics of natural intestinal endocrine cells, and can express and secrete many intestinal hormones such as GLP-1 (McCarthy et al., 2015). We used the method of CCK8 to determine the concentration of PA, berberine and GLP-1 (9-36) amide in STC-1 cells (Figure 6A). STC-1 is a cell line that can secrete GLP-1. We extracted the culture medium after cell culture for ELISA detection. We can see that the secretion of GLP-1 increases after adding berberine (Figure 6B). We used Western blot to detect the protein contents of GLP-1R, PKA, PC1/3 and PC2 in STC-1 cells (Figure 6C). After statistics, we found that proteins increased after the addition of berberine, but after the administration of GLP-1R inhibitor, this trend of berberine was inhibited (Figures 6D–G). We detected GLP-1R, PC1/3, PC2 and Pdx1 in cells by PCR, and found that their mRNA expression level increased after berberine administration (Figures 6H–K). These data indicated that berberine activated GLP-1/GLP-1R/PKA signaling pathway in STC-1 cell.

### Berberine activated GLP-1/GLP-1R/PKA signaling pathway in αTC1/6 cell

**FIGURE 7:**
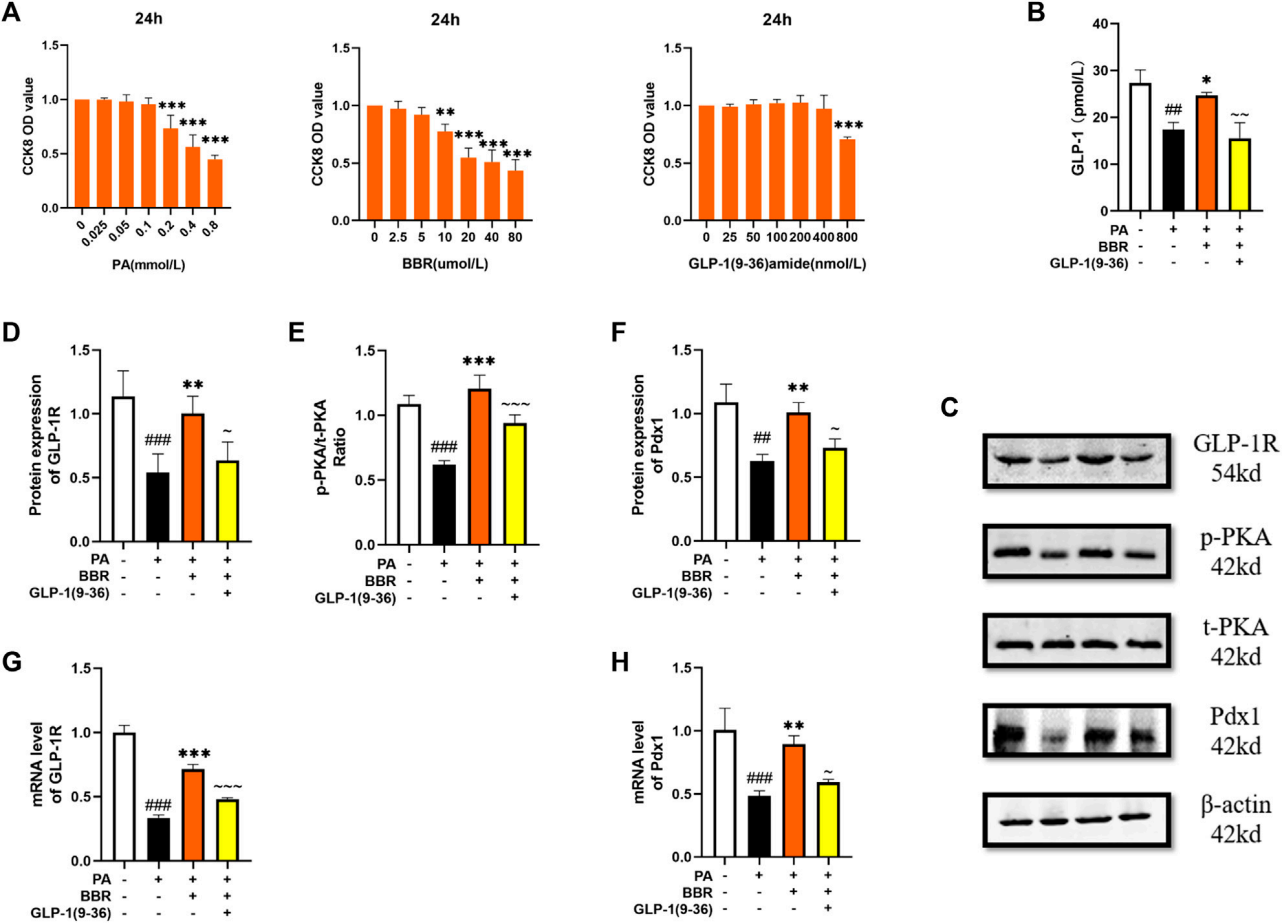
Berberine activated α GLP-1/GLP-1R/PKA signaling pathway in αTC1/6 cells. **(A)** The cck8 representative figures of PA, BBR and GLP-1 (9-36) amide (*n* = 3). **(B)** Representative figure of GLP-1 in cell (*n* = 3). **(C)** Representative western blots for immunoprecipitation of GLP-1R, PKA and Pdx1 in αTC1/6. **(D)** The quantification of GLP-1R immunoprecipitation in αTC1/6 (*n* = 4). **(E)** The quantification of PKA immunoprecipitation in αTC1/6 (*n* = 4). **(F)** The quantification of Pdx1 immunoprecipitation in αTC1/6 (*n* = 4). **(G)** The mRNA levels of GLP-1R in αTC1/6 of different groups (*n* = 3). **(H)** The mRNA levels of Pdx1 in αTC1/6 of different groups (*n* = 3). All data are presented as means ± SEM. Compared to control group, #*p* < 0.05, ##*p* < 0.01, ###*p* < 0.001; Compared to model group, *p < 0.05, **p < 0.01, ***p < 0.001; Compared to BBR group, ∼*p* < 0.05, ∼∼*p* < 0.01, ∼∼∼*p* < 0.001.

αTC1/6 cell has the physiological characteristics of islet α cell (Suga et al., 2019). We used the method of CCK8 to determine the concentration of PA, BBR and GLP-1 (9-36) amide in αTC1/6 cell (Figure 7A). We extracted the culture medium after cell culture for ELISA detection, and we can see that the secretion of GLP-1 increases after adding berberine (Figure 7B). We detected the protein contents of GLP-1R, PKA, and Pdx1 by Western blot method (Figure 7C). After statistics, we found that the expression level of these proteins increased after adding berberine, but after giving GLP-1R inhibitor, this trend of berberine was inhibited (Figures 7D–F). We also detected PC1/3 and PC2, but they were too low to be detected. We detected GLP-1R and Pdx1 in cells by PCR, and the trend was also consistent with the trend of their protein expression (Figures 7G, H). These data indicated that berberine activated GLP-1/GLP-1R/PKA signaling pathway in αTC1/6 cell.

## Discussion

In animal experiments, we verified the therapeutic effect of berberine on T2DM and the function improvement of islet β cell. Subsequently, we examined molecules of the GLP-1/GLP-1R/PKA signaling pathway in pancreatic and intestinal tissues and found that their expression was increased. Although previous studies on berberine promoting GLP-1 have been performed, they are limited to the impact on intestinal L cell. In our experiment, we took the function of α cell into consideration and observed the effect of berberine on pancreatic islet α cell, which made the evidence more convinced.

We focused on the effect of berberine on the islet cells of db/db mice. In our immunofluorescence experiment with insulin labeled β cell and glucagon labeled α cell, we found that after berberine treatment, the ratio of β cell to α cell increased. From this, we can conclude that berberine mainly affects the number and function of β cell to treat T2DM. The study indicates that the process is due to the transdifferentiation between islet cells, and the intestine-islet GLP-1/GLP-1R/PKA signal pathway is one of the important pathways to produce this process (Baggio and Drucker, 2007; Mayendraraj et al., 2022).

GLP-1 is one of the hormones responsible for the incretin effect, and exerts a wide variety of actions such as potentiation of glucose-stimulated insulin secretion, reduction of appetite, delay of gastric emptying and inhibit β cell dysfunction (Rondas et al., 2013). However, with the progress of research, it is found that GLP-1 is produced jointly by intestinal L cell and islet α cell, and acts cooperatively through endocrine and paracrine pathways, GLP-1 produced by islet α cell can act on islet β cell faster with paracrine effect (Vilsbøll, 2009; Villalba et al., 2020). With the treatment of berberine, like other studies, we have found an increase in GLP-1 secretion from intestinal L cell (Yu et al., 2015; Wang et al., 2021), but in addition, we have also noticed a significant increase in GLP-1 secretion in the pancreatic islets, which has been ignored in previous studies. From this, we can prove that berberine not only activates GLP-1R of islet β cell through the secretion of intestinal L cell, but also promotes the function of GLP-1 secretion of islet α cell.

PC1/3 and PC2, as the important proteases of β cell, play an important role in the process of transforming proinsulin into insulin (Lafferty et al., 2021). Islet specific transcription factor Pdx1 has the function of maintaining islets β cell identity, inhibiting the transformation of β cell to α cell and promoting the secretion of insulin by β cell (Ebrahim et al., 2022). We detected the expression levels of these molecules in intestinal and pancreatic tissues, and the results showed an increase in expression in both tissues. This also indicates that berberine activates the GLP-1/GLP-1R/PKA signaling pathway in intestinal and pancreatic tissues, which synergistically improves the function of pancreatic β cell.

In order to have a further study about the effect of berberine on GLP-1/GLP-1R/PKA signaling pathway, we performed experiment *in vitro*. Min6 cell, a kind of islet β cell line, has been used as a model for glucose metabolism *in vitro* (Wang and Hu, 2021). In our experiment, berberine stimulation increased the expression of related molecules in the GLP-1/GLP-1R/PKA signaling pathway in MIN6 cells. STC-1 cell is an intestinal L cell line and often used in GLP-1 related research as a cell secreting GLP-1 (Wei et al., 2020). After we administered berberine stimulation, we found an increase in GLP-1 secretion by STC-1 cells, as well as an increase in the expression of molecules such as GLP-1R, PC1/3, PC2, and Pdx1. However, the administration of GLP-1R antagonists inhibited this phenomenon, demonstrating the role of berberine in activating the GLP-1/GLP-1R/PKA signaling pathway in STC-1 cell.

We investigated the effect of berberine stimulation on the GLP-1/GLP-1R/PKA signaling pathway in αTC1/6 cell. With the discovery of the endocrine function of islet α cell in recent years, some people began to use it in some experiments (He Y. et al., 2022). As a secretory cell of GLP-1, it was stimulated by berberine and showed the same phenomenon of increased GLP-1 secretion as STC-1 cell, as well as increased expression of GLP-1R, p-PKA, Pdx1 and other related molecules. But the difference is that we did not observe a significant increase in PC1/3 and PC2 in it. A study has shown that mature α cell only express low levels of PC1/3 (Kilimnik et al., 2010), and GLP-1R agonists increase α cell PC1/3 expression through a β cell GLP-1R dependent manner (Garibay et al., 2018; Saikia et al., 2021). From this, we speculate that the significant increase in PC1/3 and PC2 that could not be detected may due to we only studied the effect of berberine alone on αTC1/6 cell. These results indicate that berberine can not only act on intestinal cells, but also promote the secretion of GLP-1 by pancreatic islet cells. The combined action of paracrine and endocrine produced GLP-1 on the GLP-1R of β cell activates its downstream signaling pathway.

Intestine-islet GLP-1/GLP-1R/PKA signaling pathway plays an important role in maintaining the identity and function of β cell. Although, there are some of studies related to the treatment of T2DM by berberine in activating this signaling pathway in intestine cell, it is few study about the paracrine effect of GLP-1 in α cell (Sandoval and D’Alessio, 2015). We consider that islet α cell is also indispensable in the process of islet function improvement. According to our research, it has been proved for the first time that berberine can improve T2DM by promoting the synergistic effect of intestinal-islet GLP-1/GLP-1R/PKA signal pathway. At the same time, we also carried out cell experiments to more clearly demonstrate that berberine promotes GLP-1 secretion and GLP-1R and its downstream molecules expression.

## Conclusion

In conclusion, we confirmed *in vivo* that berberine has the effect of treating T2DM and enhancing the function of islet β cell, and then showed that this effect is produced by activating the GLP-1/GLP-1R/PKA signal pathway in the intestine and islet. Then we verified the effect of berberine on GLP-1/GLP-1R/PKA signal pathway in related cells through *in vitro* experiments. This experiment more comprehensively revealed the mechanism of berberine acting on GLP-1/GLP-1R/PKA signal pathway, and also showed that islet α cell probably play an important role in the treatment of T2DM, providing some ideas for the follow-up study of islet function.

## Data Availability

The original contributions presented in the study are included in the article/supplementary material, further inquiries can be directed to the corresponding authors.

## Abbreviations

T2DM, Type 2 diabetes; GTT, Glucose tolerance test; ITT, Insulin tolerance test; HOMA-IR, Homeostatic model assessment of insulin resistance; FBG, Fasting blood glucose; GLP-1, Glucagon-like peptide-1; PA, Palmitic acid; BCA, Bis-creatine; RT-qPCR, Real-time quantitative polymerase chain reaction.
